# Probiotics and the Microbiome in Celiac Disease: A Randomised Controlled Trial

**DOI:** 10.1155/2016/9048574

**Published:** 2016-07-21

**Authors:** Joanna Harnett, Stephen P. Myers, Margaret Rolfe

**Affiliations:** ^1^Faculty of Pharmacy, University of Sydney, Sydney, NSW 2006, Australia; ^2^NatMed-Research, Division of Research, Southern Cross University, Military Road, Lismore, NSW 2480, Australia; ^3^University Centre for Rural Health, School of Public Health, University of Sydney, 61 Uralba Street, Lismore, NSW 2480, Australia

## Abstract

*Background*. There is limited research investigating the composition of the gastrointestinal microbiota in individuals with celiac disease (CoeD) reporting only partial symptom improvement despite adherence to a strict gluten-free diet (GFD). The aim of this research was to determine if the gastrointestinal microbiota could be altered by probiotic bacteria and provide a potential new therapy for this subgroup.* Methods*. A multicentre RCT was conducted between January and August 2011 in Australia. Participants included 45 people with CoeD reporting only partial symptom improvement despite adherence to a strict GFD for a minimum of 12 months. Participants took 5 g of VSL#*™* probiotic formulation (*n* = 23) or 5 g placebo (*n* = 22) orally twice daily for 12 weeks. The main outcome measured was the efficacy of the probiotic formula in altering faecal microbiota counts between baseline and week 12. Safety was determined by safety blood and monitoring adverse events.* Results*. SPSS*™* multivariate repeated measures analysis (95th confidence level) revealed no statistically significant changes between the groups in the faecal microbiota counts or blood safety measures over the course of the study.* Conclusion*. The probiotic formula when taken orally over the 12-week period did not significantly alter the microbiota measured in this population. The trial was registered with Australian and New Zealand Clinical Trials Register ACTRN12610000630011.

## 1. Introduction

The microbiome and its role in celiac disease (CoeD) are currently under intensive investigation. A major goal of this research is to determine the roles played by specific components of the gut microbiota in this condition and to investigate the potential for beneficial therapeutic intervention [1–10].

To date, the study populations have been primarily infants and children with CoeD [1–9]. More recently, two studies have been conducted investigating the intestinal microbiota of adults with the condition [10, 11]. A comprehensive picture of the CoeD microbiome is yet to be identified due to differing techniques employed to analyse small intestinal mucosal and luminal microbiota. In general, the majority of studies report a reduction in* Bifidobacteria* species and/or* Lactobacillus* species relative to gram negative bacteria. This specific bacterial distribution produces a cytokine profile similar to that known to be induced by the ingestion of gliadin in CoeD patients with increased interferon gamma (IFN-*γ*), tumour necrosis factor alpha (TNF-*α*), and interleukin 12 (IL-12) [12]. These findings have triggered* in vitro* investigations of potential benefits from probiotic strains.

Specific strains of* Bifidobacterium* genus,* B. bifidum* IAT-ES2 and* B. longum* ATCC 15707, have been demonstrated* in vitro* to protect against the inflammatory response and mucosal damage caused by gliadin peptides [13]. This* in vitro* study indicated that the two* Bifidobacteria* strains could exert immunoregulatory effects caused by an altered microbiota by reducing the release of the inflammatory cytokines IFN-*γ* and TNF-*α* and increasing interleukin 10 (IL-10) in peripheral blood mononuclear cells. Additionally,* B. lactis* has also been shown to contribute to mucosal protection by reducing the toxic effect of wheat gliadin on intestinal epithelial cells and small intestinal villous architecture [14].

To our knowledge only one human clinical trial has been conducted looking at the effects of probiotic supplementation in CoeD. A single strain of* B. infantis* failed to produce an improvement in intestinal permeability in active CoeD, possibly due to the limited duration of the trial which was conducted over 3 weeks [15].

The aim of this study was to examine the effects of a probiotic supplement on the CoeD microbiota. We selected the multispecies probiotic VSL#3 which has been found to hydrolyse gliadin polypeptides and reduce the toxic properties of wheat flour and was superior to other commercial available multistrain probiotics [16]. VSL#3 has also been shown to promote and maintain remission and alter the microbiota of individuals with other gastrointestinal pathologies including ulcerative colitis and pouchitis [17, 18] and stimulate the expression of the “house-keeping” molecules transforming growth factor beta-1 (TGF*β*-1) and claudin-2 in Caco-2 cells which are involved in mucosal protection [19].

## 2. Methods

### 2.1. Study Design

This multicentre study was a randomised, double-blinded, and placebo-controlled trial over 12-week period.

The study was approved by the Human Research Ethics Committee of Southern Cross University (ethics approval number ECN-10-008). The research was conducted in compliance with GCP and in accordance with the guidelines of the Australian National Health and Medical Research Council and the Declaration of Helsinki (as revised in 2004). The trial was registered with the Australian and New Zealand Clinical Trials Register (ACTRN12610000630011).

### 2.2. Study Population

The study targeted people with CoeD who were still being troubled by symptoms despite reporting adherence to a strict gluten-free diet (GFD) for the previous twelve months. Participants were recruited through the New South Wales Coeliac Association email database which invited respondents who still experienced persistent symptoms despite a GFD to contact the study coordinator. All participants received a study information sheet outlining the study and signed an informed consent form agreeing to participate.

Inclusion criteria were (a) being between 18 and 70 years of age; (b) CoeD confirmed by small bowel biopsy more than twelve months before enrolling in the study; and (c) being currently on a GFD and for at least twelve months. It was suggested post hoc that normalisation of serology (tissue transglutaminase (tTg) antibodies and/or endomysial antibodies) and/or evidence of partial or complete villous architecture repair as an additional inclusion factor would have provided greater homogeneity of the study population. Sensitivity analysis was undertaken on the inclusion criteria a priori and with the effect of this post hoc addition.

Individuals were excluded if they (a) were pregnant; (b) were less than 18 years of age; (c) were diagnosed with CoeD in the preceding 12 months; (d) were consuming a diet containing gluten; (e) were diagnosed with major gastrointestinal pathology (e.g., Crohn's disease or ulcerative colitis); (f) had short bowel syndrome; (g) had recent oral or bowel surgery; (h) had cancer, were HIV-positive, were active alcoholic, and/or had illicit drug dependence, (i) use nonsteroidal anti-inflammatory drugs, steroids, or antibiotics in the four weeks before the start of the trial; (j) had clinical abnormalities in serum urea, electrolytes, creatinine, or liver function values; and (k) were unwilling to comply with the study protocol or in the opinion of the investigators could compromise the study.

A power calculation using PASS 2008 was undertaken using data from a pilot study. It was estimated that sample sizes of 19 per group would have 80% power to detect differences between groups with a mean change of 2.5 with standard deviations of the changes of 3.0 in each group at *p* = 0.05 (1-tailed). Forty-five participants were recruited to accommodate for a possibly 20% drop-out rate.

### 2.3. Randomisation and Blinding

Participants were randomly allocated into a treatment or a placebo group. The randomisation schedule was prepared by an independent academic using a computer-generated blocked random sequence. The code was kept by the independent academic in an inaccessible locked computer file. The preparations were distributed in numerical order, matching the participants' enrolment number with the number on the intervention label. Opaque code break envelopes were produced to deal with any serious adverse effects and kept by the unit's research coordinator, who was not involved in the management of this trial, and accessed by the researchers on an as needed basis. The code was not broken until the trial was completed and the database was locked. The code was broken in two steps, firstly allocation to group A or B to allow blinded statistical analysis and secondly into actual treatment allocation on completion of the analysis.

The researchers, the participants, and the statistician were blinded to treatment allocation.

### 2.4. Study Medication

The study preparation VSL#3 [VSL Pharma, USA] was a sachet of a proprietary blend of probiotic bacteria containing 450 billion viable lyophilised bacteria* Streptococcus thermophilus*,* Bifidobacterium breve*,* Bifidobacterium longum*,* Bifidobacterium infantis*,* Lactobacillus acidophilus*,* Lactobacillus plantarum*,* Lactobacillus paracasei*, and* Lactobacillus delbrueckii* subsp.* bulgaricus.* The preparation is not listed on the Australian Register of Therapeutic Goods (TGA) and was subject to a CTN notification.

A placebo was made up with the same excipient base as the active formulation, which is maltose derived from corn, and was identical in size, weight, and packaging to the active medicine.

Study preparations were taking one sachet orally with water or juice with both the morning and evening meals for 12 weeks. Instructions were given to use noncarbonated and nonheated beverages.

### 2.5. Outcome Measures

The primary efficacy outcome measure was microbial counts and a comparison between baseline and end of the study of predominant, pathogenic, and opportunistic bacteria (colony forming units (CFUs) per gram of faeces), yeasts (parts per gram of faeces), and detection or nondetection of parasites.

Secondary efficacy outcome measures included urinary metabolomics and faecal lactoferrin.

Safety outcome measurements included safety blood (full blood count, liver function tests, and urea, creatinine, and electrolytes values) and adverse event monitoring.

All measurements were taken at baseline and at the end of the 12-week study period.

### 2.6. Microbiology

Participants collected a faecal specimen and placed it into three conical tubes; one containing formalin, another containing culture and sensitivity media, and the third conical containing a nucleic acid extraction buffer. The refrigerated specimens were sent to Metametrix*™* Laboratory (Duluth, Atlanta, GA, USA) for microscopic and molecular analysis as described by Scott et al. [20]. The target microbiota included in the analysis are presented in Table 1.

### 2.7. Urinary Metabolomics

Urinary organic acids that are products of dietary, bacterial, protozoal, or fungal metabolism in the luminal gut were measured as *μ*g/mL at baseline and again at week twelve in both treatment arms (placebo and probiotic-supplemented group) by Metametrix Laboratory (Atlanta, GA, USA).

Participants collected a first morning midstream sample after initial voiding (10–12 mL). The participants were instructed to freeze the urine specimen after collection. Women were asked not to collect specimens when menstruating. Frozen specimens were transported to the laboratory using refrigerated transport.

### 2.8. Faecal Lactoferrin

On receipt of faecal samples at Metametrix Laboratory, and before DNA-PCR analysis, faecal samples were assessed specifically for lactoferrin. The methodology employed is now proprietary to Genova Diagnostic Laboratories (Asheville, North Carolina, USA).

#### 2.8.1. Adherence to a Gluten-Free Diet (GFD): The Three-Day Diet Diary

A three-day diet diary instrument was employed to assess adherence to a GFD. Participants were asked to list what they had consumed, including foods, fluids, and medications and their brand names and how they had prepared or purchased them, that is, at home or at a food outlet, over a three-day period at baseline and during week six and week twelve. The diet diary was then analysed for sources of gluten, in conjunction with an in-depth nutritionist consultation (conducted by Joanna Harnett). If foods containing gluten were reported in the initial diet diary or in the nutritional consultation, participants were excluded. At weeks six and 12, gluten was recorded and considered a confounding factor in the analysis.

### 2.9. Safety Assessment 

#### 2.9.1. Safety Blood

Safety blood measures (full blood count, liver function test, and electrolytes, urea, and creatinine values) were undertaken at baseline and at the end of the study. Blood was collected by a trained phlebotomist and analysed by laboratories accredited with the National Association of Testing Authorities (NATA), Sonic Health Care Laboratories in Sydney (Sonic-Douglas Hanly Moir), Wollongong (Southern IML Pathology) and the North Coast of New South Wales (Sullivan & Nicolaides).

#### 2.9.2. Adverse Events

Participants were required to immediately report to the study coordinator any events, medication additions, or changes. Details of ill health events and medication changes were recorded and discussed by telephone and at the final interview. Serious ill health and severe stress, for example, hospitalisation, were grounds for withdrawal from the trial and reported as serious adverse events.

### 2.10. Compliance

All remaining sachets were returned to the study site at the final interview and counted. It was assumed that any sachets not returned had been taken. Full compliance was equivalent to one sachet taken twice daily for twelve weeks (168 sachets). Compliance was recorded as doses missed out of a total of 168 sachets.

### 2.11. Statistical Analysis

The statistical packages used were SPSS PASW®Statistics GradPack 18 and version 20 SPSS. Significance was assumed if *p* ≤ 0.05.

Basic descriptive statistics were conducted to describe the characteristics of the sample population and check the variables for any violation of the assumptions underlying the statistical techniques planned to answer the research questions. Demographic and health indicator data were analysed using independent* t-*tests, analysis of variance (ANOVA), and chi-square in order to determine the homogeneity of the allocation of participants to placebo and active treatment groups.

The primary outcome was the existence of changes in faecal microorganisms from baseline to week twelve for the two groups. Analysis of quantitative counts of predominant bacteria and phyla classes was conducted with a two-way repeated measures ANOVA, with the repeated time factor baseline and week twelve, and between subject treatment groups (probiotic or placebo), where the interaction of time and group effect was the main interest. Some variables were not normally distributed and therefore did not meet the assumptions of parametric statistical tests. If skewness was deemed to be nonnormal, that is, greater or less than zero [21], transformation of data was conducted. Where indicated, a logarithmic transformation log⁡10(*x*) was conducted before an ANOVA.

Analysis of binary data for pathogenic and opportunistic bacteria, mycology, and parasites was conducted with a Logistic Generalised Estimated Equation (GEE) with a repeated time factor baseline and week twelve and between groups (probiotic or placebo), where the interaction of time and group was the main effect.

## 3. Results

### 3.1. Demographics

Seventy-five people were screened by phone against the inclusion and exclusion criteria. This resulted in 45 in-person interviews and 45 subsequent enrolments (37 females and 8 males) between the two study sites; Sydney (*n* = 31) and Lismore (*n* = 14). The mean age of the participants at baseline was 47.3 years. The participants resided in New South Wales (*n* = 43) and the Australian Capital Territory (*n* = 2) of Australia.

### 3.2. Participant's Clinical Characteristics

All participants met the diagnostic criteria/definition of CoeD outlined by The Australian Coeliac Association. All participants reported partial symptom improvement in response to the GFD but were troubled by some residual gastrointestinal symptoms and fatigue. Symptoms were mild to moderate, as rated from a baseline validated Celiac Disease Questionnaire (CDQ) [22]. The symptoms measured included urgency to defecate, loose stools, abdominal discomfort and cramping, bloating and flatulence, incomplete defecation, eructation, and nausea.

With the exception of two participants, all participants reported the normalisation of serology. Not all participants who reported normalisation of serology after 12-month adherence to a GFD had a follow-up biopsy (see Table 2). A baseline clinical assessment determined the absence of red flags, that is, severe and persistent symptoms, unexplained weight loss or fever, blood in the stool, or black stools. All participants were under the care of a medical doctor or gastroenterologist.

*Withdrawals*. Three participants withdrew in the intervention stage of the clinical trial. Two participants from the placebo group had difficulty in complying with the study protocol and one participant in the probiotic group withdrew due to the study medication worsening their constipation.

*Serious Adverse Events*. There were three serious adverse events reported during the study, all of whom were taking placebo and each case was deemed unlikely to be associated with the study medication. One 51-year-old female had an allergic reaction resulting in angioedema at week 6 of the trial requiring oral steroids; she continued taking the study medication and reported it to the study supervisors at week 12. A 31-year-old female travelling in Europe broke her thumb and wrist and also developed pneumonia requiring hospitalisation which was reported at the end of the trial. A 61-year-old female had routine surgery soon after trial completion which was complicated by bilateral deep vein thrombosis. All data of the three participant was retained in the dataset as they had a complete set of results prior to reporting the adverse events. Furthermore, the events were deemed unlikely to be related to the placebo medication.

### 3.3. Adverse Events

VSL#3 was generally well tolerated with two participants on the placebo and two participants on the active arm reporting mild bloating.

### 3.4. Compliance

The results of an independent* t*-test showed that there was no significant difference (*p* = 0.488) between the placebo group (*n* = 21) and the active group (*n* = 21) in number of doses missed. Compliance in this study was 95.2%.

### 3.5. Final Dataset

Complete data was analysed for forty-two participants between 18 and 74 years of age (yoa), with a mean age of 47.5; 21 participants in the active group between 18 and 74 yoa, mean age of 47.1 (±16.06) years, with a mean body mass index (BMI) of 23.64 kg/m^2^; and 21 participants in the placebo group between 23 and 66 yoa, mean age of 47.5 (±12.87) years, and a mean BMI of 23.2 kg/m^2^.

The two groups did not differ significantly (*p* ≥ 0.05) in any of the comparisons measured at baseline including age, gender, medication use, family history of CoeD, degree of villous architecture recovery, food intolerance, and early childhood microbiome influences (Table 2).

Forty participants reported partial to full normalisation of serology (*n* = 30) or villous architecture repair (*n* = 10) prior to the study commencing. One participant in the active group and one participant in the placebo group reported persistent villous atrophy accompanied by mild to moderate gastrointestinal symptoms.

A sensitivity analysis for homogeneity was conducted by removing the two participants with persistent villous atrophy. The removal of their data did not alter any of the comparative microbiota reported.

### 3.6. Predominant, Opportunistic, and Pathogenic Bacteria

Descriptive statistics for the predominant bacteria showed that only* Streptomyces* sp. (*p* = 0.058) differed between the two groups at baseline and week 12. At week 12 only,* Mycoplasma* sp. (*p* = 0.026) differed between the two groups (Table 3). The results of repeated measure ANOVA showed a significant change in the counts of* Streptomyces* sp. (*p* = 0.02) by the treatment effect (Table 4). The bacterial species* Bifidobacteria* (*p* = 0.001) showed a significant reduction and* Escherichia coli* (*p* = 0.005) showed a significant increase over time. Estimated means and results of Bonferonni-adjusted pairwise comparisons showed a significant time response of the predominant bacteria* Bifidobacteria* sp. and* Escherichia coli* (Table 5).

Generalised Estimated Equations (GEE) showed a decrease in the detection rate of* H. pylori* over time in both groups to be close to reaching significance (*p* < 0.08) (Table 6).

There was no significant difference for our principal comparison of interest, the time by treatment effect for any predominant (Table 4), opportunistic, or pathogenic bacteria measured (Table 7).

### 3.7. Mycology

Results of the cross tabulations showed that the probiotic group had significantly higher prevalence of* Saccharomyces* sp. at baseline (*p* = 0.02) than the placebo group (Table 6). The results of the logistic GEE for mycology showed that* Saccharomyces* sp. counts reduced significantly with treatment (*p* = 0.04). There was no significant difference by time and treatment for any fungi measured (Table 7).

### 3.8. Parasites

There was no difference between groups in the detection rate of parasites (Table 6). The logistic GEE results showed the general parasite incidence (*p* = 0.002) and parasites with an unknown taxonomy (*p* = 0.01) reduced significantly over time (*p* < 0.016) in both groups with no significant difference for treatment or time for any parasites measured (Table 7).

### 3.9. Urinary Organic Acids

A significant time by treatment effect for the reduction of urinary D-lactate measures (*p* = 0.004) was observed between groups at week 12 with a decrease in the probiotic group compared to the placebo.

### 3.10. Blood Safety Parameters

A statistically significant time by treatment and treatment effect was observed for blood urea levels. Analysis for outliers showed skewing by one participant's urea result being elevated at both baseline and week 12; therefore, this was unlikely to be a group effect. Sensitivity analysis with this participant excluded did not yield any statistical significance.

## 4. Discussion

This is the first human study to have measured the microbiological effects of a multiple species gram-positive probiotic in individuals with CoeD. The results demonstrated no significant differences between the active and placebo groups in the primary outcome measure and faecal microflora counts.

These results differed significantly from the positive outcomes reported in other conditions measuring the effect of VSL#3 on the microbiota of patients with ulcerative colitis [23] and pouchitis [24].

The results of the gastrointestinal microbiome reported in this study were supported by the results of a secondary outcome measure that evaluated the participant's symptoms and quality of life. The questions included in the validated CoeD specific questionnaire developed by Häuser and colleagues [22] are presented in the Appendix. No clinically significant improvement in symptoms was observed between groups. This finding will be reported in detail elsewhere. Briefly here, scores were calculated using scores obtained from participants' self-reporting. Participants were asked to score twenty-eight questions on a scale of 1–7 at baseline and at weeks four, eight, twelve, and 16 of the study period. The questions were categorised into four subscales (emotion, worry, and social and gastrointestinal symptoms). The four subscales' scores were then calculated to provide a score for the individual areas of interest. The collective total score of the four subscales was calculated at baseline and at week twelve.

A number of potential limitations need to be considered when interpreting the results of this study including population homogeneity, laboratory methodology, study medication, dose and duration of therapy, blinding, microbial survival, and potential placebo interference. Each of these will be discussed briefly.

It is known that that causes of persistent symptoms in treated CoeD are relatively heterogeneous [25]. A recent meta-analysis reported 30% of individuals with CoeD reported IBS-type symptoms despite adherence to a GFD [26]. Furthermore, altered duodenal microflora has been proposed as a potential cause of persistent symptoms [10], and that treatment with probiotics and/or prebiotics could be useful [27]. Nevertheless, the homogeneity of this study may have been compromised through our global treatment approach and the inclusion of participants whose residual symptoms may have been from multiple nonserious causes. Based on the evidence available at the time this study was conducted, it was considered that probiotics may play a beneficial role in the general causes of mild-moderate persistent symptoms in individuals with CoeD [25, 28–30]. Probiotics have been demonstrated to be beneficial in small intestinal bacterial overgrowth [31]; lactose intolerance [32]; pancreatic insufficiency [33]; poorly absorbed short chain carbohydrates [34]; intestinal permeability [35]; and irritable bowel syndrome [36]. Future studies could consider each of these causes as a specific subgroup. The homogeneity of the study population may have been further compromised by relying on self-reporting of serology and biopsy results following adherence to a GFD.

Transportation of specimens is often raised as a potential methodological concern. Poor transportation practices may result in the continued growth of organisms, which in turn may lead to a significant change in the balance of microbes present. Many aerobes will grow at the expense of anaerobes in nutrient broth transport media. This limitation was overcome in this present study by using formalin and nucleic acid extraction buffer which prevents continued growth of organisms [20], thus improving the capacity to detect and quantify anaerobic bacteria.

A contribution from this study was the analysis of yeasts and fungi and parasites. A real limitation of this present study was that the laboratory assessment of faecal specimens did not include counts of* Streptococcus* sp. and* Enterococcus* sp. as the laboratory had not established algorithms for the quantitative computation of these microorganisms. This resulted in not being able to report the recoverable numbers of* Streptococcus* sp. which is a constituent of VSL#3.

A variety of molecular techniques have been employed to explore the bacterial communities in the gastrointestinal tracts of individuals with CoeD [3, 4, 37] yielding similar results to our baseline measures. The analysis of both baseline and follow-up specimens was conducted as one batch to improve scientific rigor and reduce any risk of bias. As with all methodologies, it is important to consider the validity of the results reported against the specificity and experimental variability of the method employed [38]. The scientific rigor of this study could have been further enhanced by having a second laboratory replicate and validate the laboratory methods on a random set of samples.

A potential limitation is nonviability of the study medication. We undertook a range of quality assurance process to ensure that this was unlikely including verification of our randomisation coding; independent laboratory analysis of biological viability of the active probiotic and the placebo; and verification that manufacturers, transporters, and participants had stored the study medication in refrigerated conditions.

There are few human studies that have measured the faecal microbial composition changes in response to the administration of VSL#3. In the prevention of pouchitis, one study reported a significant increase in* lactobacilli, Bifidobacteria*, and* S. thermophilus* after 6 months of treatment of 900 billion CFUs per day of VSL#3 that correlated with improvement in other disease indices [39]. This was at the same dose as used in this study but double the duration. Three studies in ulcerative colitis have demonstrated positive changes in disease indices after administration of VSL#3 [40–42]. Two administered 3600 billion CFUs per day for 8 weeks and one 1800 CFUs for 12 weeks. All of these studies used double to four times the dosage used in our study which is an important factor to consider in future research.

Since the completion of this study, the literature regarding the efficacy of different treatment periods for probiotics has emerged. Ritchie and Romanuk's [43] meta-analysis reported efficacy for treatments of one to two weeks, three to four weeks, and five to eight weeks, with treatments of nine to 24 weeks having significantly higher efficacy than those of three to four weeks. These findings were supportive of our earlier decision to give VSL#3 for a 12-week period.

Sachets of the study materials were opened by the study coordinator (Joanna Harnett) at the end of the study and the appearance of the powders differed in colour and texture. These changes may or may not have been there since the commencement of the study and we recommend that other researchers using probiotics seek quality assurance of the identical nature of the active and placebo and any changes over time from the manufacturer. While we considered that it was unlikely that participants would be aware of any differences between the active and placebo powders as they were dispensed in individual sachets, we are aware that these differences affect the veracity of the blinding procedure.

It is perplexing as to why no difference in the faecal counts of the* Bifidobacteria* and* Lactobacillus* species between groups was observed. It is plausible that the probiotic species did not survive the physiological environment of the upper gastrointestinal tract [44]. Viable counts of bacteria need to make their way to the small and large intestine, where they exert their beneficial effects [45]. The buffering capacity of food on the survival of probiotic microbes during gastrointestinal transit has been proposed suggesting that nonenteric coated bacterial probiotic products should be taken with or just before a meal and that the meal should contain some fats [46]. We did not control fat content in this study which should be considered in future research. Further studies would be advised to consider trialling enteric coated probiotic formulas with meals containing fat to aid in circumventing digestive acids and enzymes.

Another possible explanation for seeing no difference in the faecal microbial counts between groups, while largely speculative, is that the placebo may have exerted an effect on the microbiological measures. Both groups received maltose, as either 6 g in the placebo arm or 2.4 g maltose as an excipient in the active arm. Energy sources enable a complex cycle of cross-feeding, growth, and metabolic activity. Maltose is a disaccharide and technically a food source, and theoretically any microbiological changes it may have caused could have masqueraded as a time effect in both groups, rather than a time by treatment effect. Individuals with CoeD frequently have a transient disaccharidase lactase deficiency [25]; however, less was known regarding disaccharidase maltase deficiency in this population. Since the completion of this trial, children with CoeD with intact villi were shown to have significant disaccharidase deficiencies, including a maltase deficiency [47]. Although a number of positive outcome RCTs have been published using VSL#3 using maltose as a placebo, it is important to note that these studies were in patient populations who have not been reported as having a higher prevalence of disaccharidase deficiencies [41, 48, 49].

Paradoxically, all three bacterial species of VSL#3 are major producers of lactic acid; yet their administration for twelve weeks appears to have led to a decrease in lactate. Lactate accumulates only when there is fast fermentation. If substrates are fermented slowly, D-lactate is converted into beneficial short chain fatty acids (SCFAs) [50]. One possible explanation is that VSL#3 changed the fermentation rate of the intestinal milieu but we are able to neither attribute that change to a specific organism nor prove it. Further studies should consider measuring changes to faecal SCFAs. Another possible explanation is that there was a D-lactate-producing organism, which we did not measure, reducing in numbers in response to the administration of the probiotic.

Decreases in Faecal* H. pylori* over time across the entire group nearly reached significance. This suggests that perhaps some participants may have had a transient infection. Transient* H. pylori* infection with spontaneous resolution, without intervention, has been described [51]* H. pylori* is the most successful human pathogen, infecting an estimated 50% of the global population [51].* H. pylori* is rightly classified as a formidable pathogen and is the first bacterium to be classified as a carcinogen by the World Health Organisation: it infects up to half the world's population, although disease is the exception rather than the rule [52]. In a clinical setting, further information regarding the significance of the faecal detection of* H. pylori* would be obtained from the results of a urea breath test and faecal antigens [51] which may be valuable additions to a research protocol.

## 5. Conclusion

VSL#3 did not significantly change the gastrointestinal microbial counts measured in a subgroup of individuals with CoeD with persistent symptoms at 900 CFUs over 12 weeks. Future research should look at increasing the dosage and duration to determine if these factors were responsible for this negative result. Further research is required to understand whether the reductions observed in urinary D-lactate have any specific metabolomic significance.

## Figures and Tables

**Figure 1:**
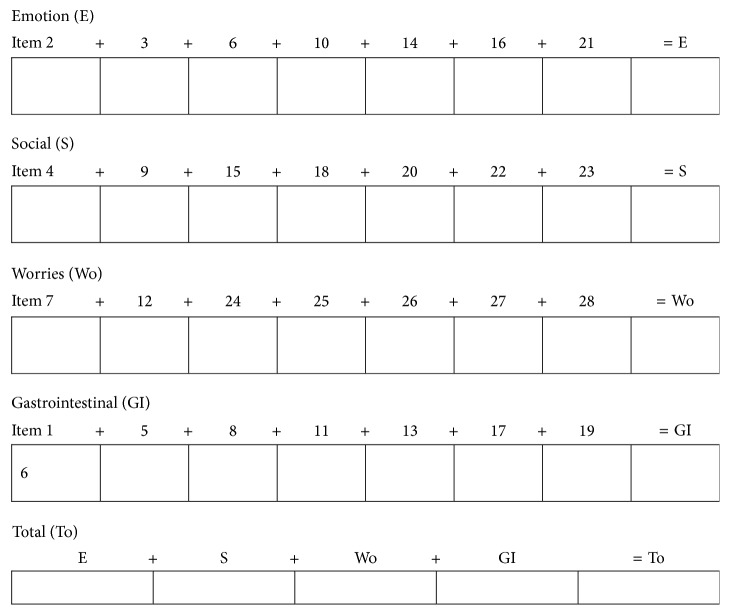


**Table 1:** Targeted microbiota.

Predominant bacteria	Opportunistic bacteria and fungi	Pathogenic bacteria	Parasites
*Bacteroides *sp.	*Achromobacter*	*Helicobacter *sp.	*Blastocystis hominis*
*Clostridia *sp.	*Alcaligenes *sp.	*Campylobacter *sp.	*Cryptosporidium *sp.
*Prevotella *sp.	*Aeromonas *sp.	*Clostridium difficile*	*Entamoeba *sp.
*Fusobacteria *sp.	*Bacillus *sp.	*E. coli *H1:O157	*Entamoeba histolytica*
*Streptomyces *sp.	*Citrobacter *sp.		*Entamoeba coli*
*Mycoplasma *sp.	*Enterobacter *sp.		*Dientamoeba fragilis*
*Lactobacillus *sp.	*Klebsiella oxytoca*		*Endolimax nana*
*Bifidobacterium *sp.	*Klebsiella pneumoniae*		*Trichomonas *sp.
*Escherichia coli*	*Morganella morganii*		*Giardia intestinalis*
	*Pseudomonas *sp.		*Ascaris lumbricoides*
	*Salmonella *sp.		*Enterobius vermicularis*
	*Staphylococcus aureus*		*Necator americanus*
	*Saccharomyces *sp.		*Strongyloides *sp.
	*Candida albicans*		*Taenia solium*
	*Geotrichum *sp.		*Trichuris trichiura*
	*Rhodotorula *sp.		*Schistosoma mansoni*
			*Clonorchis sinensis*

**Table 2:** Comparison of demographic health indicators.

Demographic and health indicators *n* (%)	Placebo (*n* = 21)	Probiotic (*n* = 21)	*p* value
Age mean (±SD)	47.5 (±12.87)	47.1 (±16.06)	0.924
Gender: males	4 (18)	3 (14)	0.946
Gender: females	17 (77)	18 (85)	0.946
Proton pump inhibitor use	4 (18)	4 (19)	0.271
Antidepressant use	4 (18)	3 (14)	0.644
Anti-inflammatory use	2 (9)	2 (9)	0.964
No family history of CD	13 (59)	16 (76)	0.474
Family history of CD	9 (40)	7 (33)	0.474
No improvement of villous architecture >12 months of GFD	1 (4)	1 (4)	0.975
Partial improvement of villous architecture after >12 months of GFD	8 (36)	12 (57)	0.975
Full villous architecture restoration after >12 months of GFD	5 (22)	5 (23)	0.975
No follow-up biopsy conducted	5 (22)	5 (23)	0.975
Having a tonsillectomy	11 (50)	12 (57)	0.843
Breast-feeding for 6–12 months	8 (36)	8 (38)	0.887
>3 course of antibiotics in first 12 months of life	13 (59)	13 (61)	0.975
>3 respiratory infections in first 12 months of life	13 (59)	15 (71)	0.975
Known food intolerance other than gluten	11 (50)	12 (57)	0.843
No known food intolerance other than gluten	11 (50)	11 (52)	0.887

**Table 3:** Descriptive statistics for the counts of predominant bacteria between treatment groups by measurement occasion.

Predominant bacterial species measures	Week	Probiotic group: counts of CFU/g faeces					Placebo group: counts of CFU/g faeces				
		*n*	Mean	SD	Min	Max	*n*	Mean	SD	Min	Max
*Bacteroide*s sp.	0	23	4.1	2.72	1.7	11.9	22	4.0	2.29	1.5	8.0
	12	21	5.6	5.39	1.9	24.0	21	4.6	2.57	1.7	12.4

*Clostridia* sp.	0	23	4.4	2.09	1.8	9.1	22	3.8	1.62	1.9	7.4
	12	21	4.4	2.93	1.6	10.7	21	4.6	2.42	1.4	9.9

*Prevotella* sp.	0	23	4.2	2.59	1.5	13.6	22	3.2	1.67	1.6	7.4
	12	21	3.3	1.73	1.7	8.5	21	2.9	1.33	1.7	5.8

*Fusobacteria* sp.	0	23	4.5	3.51	1.9	13.2	22	4.3	3.06	1.7	13.4
	12	21	4.9	7.18	1.4	34.8	21	3.8	2.84	1.3	10.2

*Streptomyce*s sp.	0	23	3.9	1.98	1.7	9.9	22	3.0	1.34	1.6	6.7
	12	21	4.1	2.15	1.5	9.2	21	3.2	2.04	1.7	9.8

*Mycoplasma *sp.	0	23	3.3	1.34	1.6	6.4	22	3.4	1.73	1.7	8.3
	12	21	4.3	2.46	1.9	9.8	21	2.9	1.13	1.6	5.4

*Lactobacillus *sp.	0	23	4.3	2.30	1.8	11.3	22	6.5	6.41	1.8	29.8
	12	21	4.6	3.48	1.5	17.3	21	6.2	6.63	2.1	32.0

*Bifidobacteria* sp.	0	23	5.2	2.91	2.2	11.8	22	5.1	2.41	1.8	9.5
	12	21	4.8	3.22	1.9	15.2	21	3.4	1.45	1.5	7.6

*Escherichia coli* sp.	0	23	3.7	1.92	1.6	8.3	22	3.3	1.44	1.6	6.4
	12	21	5.0	2.86	1.5	11.7	21	4.0	2.36	1.7	10.2

Predominant bacterial total counts	0	23	38.06	8.95	23.3	58.7	22	36.8	10.75	23.6	75.3
	12	21	41.34	15.35	22.3	73.4	21	36.0	12.18	24.5	68.0

A count of 4.1 = 4.1 E7 CFU/g is read as 41 million colony forming units per gram of faeces.

**Table 4:** ANOVA predominant bacteria results, *p* values (log_10_ transformed data).

Predominant bacteria	Treatment	Time	Time by treatment
*Bacteroides* sp.	0.686	0.198	0.992
*Clostridia* sp.	0.831	0.589	0.162
*Prevotella* sp.	0.279	0.800	0.279
*Fusobacteria* sp.	0.772	0.270	0.806
*Streptomyces* sp.	0.022	0.794	0.990
*Mycoplasma* sp.	0.097	0.719	0.106
*Lactobacillus* sp.	0.239	0.880	0.656
*Bifidobacteria* sp.	0.245	0.001	0.137
*Escherichia coli* sp.	0.235	0.051	0.516
Total counts of predominant bacterial sp.	0.252	0.877	0.458

**Table 5:** Bonferroni-adjusted pairwise comparisons of predominant bacteria measures that changed significantly with time.

Predominant bacteria	Week	Mean	SD	Lower CI (95%)	Upper CI (95%)	Multiple comparison
*Bifidobacteria* sp.	1	5.40 E7	0.412	0.443	2.109	1 vs 12 *p* = 0.004
	12	4.12 E7	0.386	3.340	4.904	

*Escherichia coli*	1	3.662 E7	0.265	3.129	4.195	1 vs 12 *p* = 0.036
	12	4.551 E7	0.405	3.713	5.349	

Mean counts expressed as E7 CFU/g (e.g., 5.40 E7 is read as 54 million colony forming units per gram of faeces).

**Table 6:** Cross tabulations: detection rate of parasites, pathogenic bacteria, opportunistic bacteria, and fungi between treatment groups by measurement occasions.

Parasite	Week	Probiotic group		Placebo group		Chi-square fisher exact test
		Not detected *n* (%)	Detected *n* (%)	Not detected *n* (%)	Detected *n* (%)	
General parasite	0	2 (8.7)	21 (91.3)	2 (9.1)	20 (90.9)	0.679
	12	5 (23.8)	16 (76.2)	6 (28.6)	15 (71.4)	0.500

Parasite unknown taxonomy	0	3 (13)	20 (87)	2 (9.1)	20 (90.9)	0.522
	12	7 (33.3)	14 (66.6)	8 (38.1)	13 (61.9)	0.500

*Blastocystis hominis*	0	20 (87)	3 (13)	17 (77.3)	5 (22.7)	0.324
	12	20 (95.2)	1 (4.8)	17 (81)	4 (19)	0.172

*Dientamoeba* sp.	0	22 (95.7)	1 (4.3)	21 (95.5)	1 (4.5)	0.744
	12	21 (100)	0 (0)	20 (95.2)	1 (4.8)	0.500

*Necator americanus *(Hookworm)	0	21 (91.3)	2 (8.7)	21 (95.5)	1 (4.5)	0.517
	12	19 (90.5)	2 (9.5)	20 (95.2)	1 (4.5)	0.500

*Trichuris* sp. (Whipworm)	0	21 (91.3)	2 (8.7)	22 (100)	0 (0)	0.256
	12	19 (90.5)	2 (9.5)	21 (100)	0 (0)	0.244

*Enterobius vermicularis* (Pinworm)	0	22 (95.7)	1 (4.3)	19 (86.4)	3 (13.6)	0.287
	12	21/100	0/0	19/90.5	2 (9.5)	0.244

*H. pylori*	0	16 (69.6)	7 (31.4)	12 (54.5)	10 (45.5)	0.398
	12	16 (76.2)	5 (32.8)	15 (71.4)	6 (29.6)	0.533

*Escherichia haemorrhagic coli*	0	20 (87.0)	3 (13)	21 (95.5)	1 (4.5)	0.405
	12	18 (85.7)	3 (13.3)	21 (100)	0 (0)	0.357

*Streptococcus* sp.	0	1 (4.5)	21 (95.5)	0 (0)	20 (100)	0.524
	12	0 (0)	21 (100)	1 (5.3)	18 (94.7)	0.475

*Enterococcus* sp.	0	4 (19)	17 (81)	3 (15)	17 (85)	0.529
	12	9 (47.4)	10 (52.6)	9 (50)	9 (50)	0.567

*Aeromonas* sp.	0	23 (100)	0 (0)	21 (95.5)	1 (4.5)	0.489
	12	20 (95.2)	1 (4.8)	21 (100)	0 (0)	0.500

*Klebsiella oxytoca*	0	22 (95.7)	1 (4.3)	22 (100)	0 (0)	0.511
	12	21 (100)	0 (0)	21 (100)	0 (0)	UAC

*Achromobacter* sp.	0	22 (95.7)	1 (4.3)	22 (100)	0 (0)	0.511
	12	21 (100)	0 (0)	19 (90.5)	2 (9.5)	0.500

*Bacillus* sp.	0	23 (100)	0 (0)	20 (90.9)	2 (9.1)	0.335
	12	21 (100)	0 (0)	21 (100)	0 (0)	UAC

*Morganella morganii*	0	22 (95.7)	1 (4.3)	21 (95.5)	1 (4.5)	0.368
	12	20 (95.2)	1 (4.8)	21 (100)	0 (0)	0.500

*Citrobacter* sp.	0	22 (95.7)	1 (4.3)	22 (100)	0 (0)	0.511
	12	21 (100)	0 (0)	21 (100)	0 (0)	UAC

Total opportunistic bacteria detected	0	20 (87)	3 (13)	18 (81.8)	4 (18.2)	0.474
	12	18 (85.7)	3 (14.3)	19 (90.5)	2 (9.5)	0.500

*Candida* sp.	0	18 (78.3)	5 (21.7)	12 (54.5)	9 (40.9)	0.190
	12	17 (81)	4 (19)	13 (61.9)	8 (38.1)	0.306

*Sacharromyces *sp.	0	19 (82.6)	4 (17.4)	11 (50)	11 (50)	0.020
	12	17 (81)	4 (19)	14 (66.7)	7 (33.3)	0.242

UAC denotes “unable to calculate” due to the specified organisms not being present in any participants in one or both treatment arms.

**Table 7:** GEE *p* values for the opportunistic and pathogenic bacteria, fungi, and parasites.

Bacteria	Treatment	Time	Time by treatment
Total opportunistic bacteria	0.95	0.65	0.52
*Morganella morganii*	UAC	UAC	UAC
*Citrobacter*	UAC	UAC	UAC
*Aeromonas* sp.	0.98	UAC	UAC
*Klebsiella pneumonia*	UAC	UAC	UAC
*Staphylococcus* sp.	UAC	UAC	UAC
*Achromobacter* sp.	0.97	UAC	UAC
*Klebsiella oxytoca*	UAC	UAC	UAC
*Helicobacter pylori*	0.42	0.08	0.59
Enterohemorrhagic* E. coli*	UAC	UAC	UAC
*Candida *sp.	0.06	0.53	0.88
*Saccharomyces *sp.	**0.04**	0.41	0.34
*General Parasite DNA detected with known taxonomy*	0.830	**0.016**	0.841
*Blastocystis hominis*	0.211	0.137	0.343
*Dientamoeba *sp.	UAC	UAC	UAC
*Parasite taxonomy unavailable*	0.892	**0.002**	0.514
*Trichuris trichiura*	UAC	UAC	UAC
*Enterobius vermicularis*	UAC	UAC	UAC
*Necator americanus*	0.583	0.094	0.565

UAC denotes “unable to calculate” due to the specified organisms not being present in any participants in one or both treatment arms.

## Appendix

### The Celiac Disease Questionnaire CDQ: Health-Related Quality of Life Index for Adult Patients with Celiac Disease

This questionnaire has been developed to find out how you have been feeling during the last two weeks. You will be asked about symptoms related to your celiac disease, your general well-being, and your mood. The questionnaire includes 28 questions. Each question offers seven possible answers ranked (1) to (7). Please read each question carefully and tick the answer that best describes how you felt during the past two weeks. If an item does not apply to you (e.g., sexual activity), please leave the question unanswered.

(1) How many times during the past two weeks was your life affected by a sudden urge to visit a bathroom for a bowel movement?

1. All of the time
2. Most of the time
3. A good bit of the time
4. Some of the time
5. A little of the time
6. Hardly any of the time
7. None of the time

(2) How often during the last two weeks did you feel physically exhausted or fatigued?

1. All of the time
2. Most of the time
3. A good bit of the time
4. Some of the time
5. A little of the time
6. Hardly any of the time
7. None of the time

(3) How often during the last two weeks have you felt frustrated, impatient, or restless?

1. All of the time
2. Most of the time
3. A good bit of the time
4. Some of the time
5. A little of the time
6. Hardly any of the time
7. None of the time

(4) How many times during the last two weeks did you refuse or avoid an invitation for dinner with friends or relatives due to your celiac disease?

1. All of the time
2. Most of the time
3. A good bit of the time
4. Some of the time
5. A little of the time
6. Hardly any of the time
7. None of the time

(5) How often during the last two weeks have your bowel movements been loose?

1. All of the time
2. Most of the time
3. A good bit of the time
4. Some of the time
5. A little of the time
6. Hardly any of the time
7. None of the time

(6) How much intellectual energy did you have during the last two weeks?

1. No energy at all
2. Very little energy
3. Little energy
4. Some energy
5. A moderate amount of energy
6. Lots of energy
7. I was full of energy

**(**7) How many times during the last two weeks were you concerned that your children could inherit or may have inherited your celiac disease?

1. All of the time
2. Most of the time
3. A good bit of the time
4. Some of the time
5. A little of the time
6. Hardly any of the time
7. None of the time

(8) How many times during the last two weeks have you been troubled by cramps in your abdomen?

1. All of the time
2. Most of the time
3. A good bit of the time
4. Some of the time
5. A little of the time
6. Hardly any of the time
7. None of the time

(9) Did you encounter any difficulties with recreational activities or sports due to your celiac disease during the last two weeks?

1. Extreme difficulties, no activities possible
2. Very considerable difficulties
3. Considerable difficulties
4. Some difficulties
5. Minor difficulties
6. Hardly any difficulties
7. No difficulties, celiac disease did not affect my recreational activities or sports

(10) How often during the last two weeks did you feel depressed or discouraged?

1. All of the time
2. Most of the time
3. A good bit of the time
4. Some of the time
5. A little of the time
6. Hardly any of the time
7. None of the time

(11) How many times during the last two weeks did you suffer from bloating or flatulence?

1. All of the time
2. Most of the time
3. A good bit of the time
4. Some of the time
5. A little of the time
6. Hardly any of the time
7. None of the time

(12) People with celiac disease often have worries and fears related to their disease. How many times during the last two weeks did you worry about or were afraid of getting cancer as a result of your celiac disease?

1. All of the time
2. Most of the time
3. A good bit of the time
4. Some of the time
5. A little of the time
6. Hardly any of the time
7. None of the time

(13) How many times during the last two weeks were you affected by a feeling of incomplete bowel evacuation?

1. All of the time
2. Most of the time
3. A good bit of the time
4. Some of the time
5. A little of the time
6. Hardly any of the time
7. None of the time

(14) How often during the last two weeks have you felt relaxed and free of tension?

1. None of the time
2. Hardly any of the time
3. A little of the time
4. Some of the time
5. A good bit of the time
6. Most of the time
7. All of the time

(15) How many times during the last two weeks did you feel isolated from or excluded by others due to your celiac disease?

1. All of the time
2. Most of the time
3. A good bit of the time
4. Some of the time
5. A little of the time
6. Hardly any of the time
7. None of the time

(16) How much of the time during the last two weeks have you felt tearful or upset?

1. All of the time
2. Most of the time
3. A good bit of the time
4. Some of the time
5. A little of the time
6. Hardly any of the time
7. None of the time

(17) How many times during the last two weeks did you suffer from repeated belching?

1. All of the time
2. Most of the time
3. A good bit of the time
4. Some of the time
5. A little of the time
6. Hardly any of the time
7. None of the time

(18) To what extent did your celiac disease restrict your sexual activity during the last two weeks?

1. No sex due to celiac disease
2. Considerable restraint due to celiac disease
3. Moderate restraint due to celiac disease
4. Some restraint due to celiac disease
5. Little restraint due to celiac disease
6. Almost no restraint due to celiac disease
7. No restraint due to celiac disease

(19) How many times during the last two weeks did you suffer from nausea or retching?

1. All of the time
2. Most of the time
3. A good bit of the time
4. Some of the time
5. A little of the time
6. Hardly any of the time
7. None of the time

(20) How many times during the last two weeks did you feel that important people such as members of your family or friends showed a lack of understanding for your celiac disease?

1. All of the time
2. Most of the time
3. A good bit of the time
4. Some of the time
5. A little of the time
6. Hardly any of the time
7. None of the time

(21) How satisfied, happy, or pleased have you been with your personal life you during the last two weeks?

1. Very unsatisfied, mostly unhappy
2. Generally unsatisfied, unhappy
3. Somewhat unsatisfied, unhappy
4. Generally satisfied, pleased
5. Most of the time satisfied, happy
6. Most of the time very satisfied, happy
7. Very satisfied, could not be happier or more pleased

(22) How many times during the last two weeks did you feel that colleagues or superiors showed a lack of understanding for your celiac disease?

1. All of the time
2. Most of the time
3. A good bit of the time
4. Some of the time
5. A little of the time
6. Hardly any of the time
7. None of the time

(23) How many times during the last two weeks did you feel limited in your professional training or career by your celiac disease?

1. All of the time
2. Most of the time
3. A good bit of the time
4. Some of the time
5. A little of the time
6. Hardly any of the time
7. None of the time

(24) How many times during the last two weeks did you feel burdened by the expenses and time required obtaining gluten-free food?

*Modified.* How many times during the last two weeks did you feel burdened by difficulties obtaining gluten-free food?

1. All of the time
2. Most of the time
3. A good bit of the time
4. Some of the time
5. A little of the time
6. Hardly any of the time
7. None of the time

(25) How many times during the last two weeks did you feel burdened by problems with your health or pension insurance provider regarding meeting the costs of gluten-free food or other celiac therapies?

*Modified.* How many times during the last two weeks did you feel burdened by problems regarding financing (e.g., costs, prescription, and reimbursement) gluten-free food or other celiac therapies?

1. All of the time
2. Most of the time
3. A good bit of the time
4. Some of the time
5. A little of the time
6. Hardly any of the time
7. None of the time

(26) How many times during the last two weeks did you experience lack of expertise regarding celiac disease from your doctors?

1. All of the time
2. Most of the time
3. A good bit of the time
4. Some of the time
5. A little of the time
6. Hardly any of the time
7. None of the time

(27) How many times during the last two weeks did you worry that your celiac disease was diagnosed too late?

1. All of the time
2. Most of the time
3. A good bit of the time
4. Some of the time
5. A little of the time
6. Hardly any of the time
7. None of the time

(28) How many times during the last two weeks did you suffer from fear of medical examinations in relation to your celiac disease, for example, blood withdrawal or enteroscopy?

1. All of the time
2. Most of the time
3. A good bit of the time
4. Some of the time
5. A little of the time
6. Hardly any of the time
7. None of the time

*CDQ-Realization and Interpretation*

*Instructions to the Investigator and the Patient.* The CDQ is presented to the patient that he can find the instructions of how to answer the questions at the beginning of the questionnaire. It takes about 10 minutes to answer the questions. The patient should be instructed to answer the questions by himself and not to ask any other persons such as partners. If the patient has completed the CDQ, the investigator should control that all questions are answered and ask if the patient has any questions regarding the CDQ and its interpretation.

*Interpretation of the CDQ*. The subscale scores result from an addition of the items of the respective subscale.  Figure 1 can be used to calculate the scores.

*Test Interpretation*. The subscale scores range between 0 and 49 in each subscale. The total score ranges between 0 and 196. High scores are indicative of a high health-related quality of life; low scores indicate a reduced health-related quality of life. Up to 1 missing item of each subscale can be substituted by the individual median of the other items of the subscale. If more than 1 item of one scale is not answered, the scores cannot be used for scientific studies. Gender differences (women with celiac disease as well as women in the general population report lower HRQOL than men, Häuser et al. [22]) should be considered. In case of group comparisons and different sex ratios compared to the German sample, the scores should be adjusted by multiple regression analysis. If multiple measurements (such as those in intervention studies) are performed, differences of ≥12 within the total score and ≥3 within each subscore can be regarded to be a minimum important clinical difference for intraindividual comparisons. For comparisons of groups, changes of ≥1/2 standard deviation can be regarded to be a minimum important clinical difference [53].

*Copyright*. The use of the CDQ for clinical studies without license fees is possible after written confirmation of the authors of the CDQ. Requests should be send to whaeuser@klinikum-saarbruecken.de.

*Literature*. See [22, 53].

## List of Abbreviations

ANOVA: Analysis of variance
CDQ: Celiac Disease Questionnaire
CoeD: Celiac disease
CFUs: Colony forming units
GA: Georgia
GEE: Generalised Estimated Equations
GFD: Gluten-free diet
IL: Interleukin
IFN: Interferon
NA: Not Available
NATA: National Association of Testing Authorities
NSW: New South Wales
RCT: Randomised Controlled Trial
TGF: Transforming growth factor
TNF: Tumour necrosis factor
UAC: Unable to calculate.
